# Sick leave due to musculoskeletal pain: determinants of distinct trajectories over 1 year

**DOI:** 10.1007/s00420-019-01447-y

**Published:** 2019-06-04

**Authors:** David M. Hallman, Andreas Holtermann, Martin Björklund, Nidhi Gupta, Charlotte D. Nørregaard Rasmussen

**Affiliations:** 1 Department of Occupational Health Sciences and Psychology, Centre for Musculoskeletal Research, University of Gävle, Kungsbäcksvägen 47, 80176 Gävle, Sweden; 2 National Research Centre for the Working Environment, Copenhagen, Denmark; 3 Department of Community Medicine and Rehabilitation, Umeå University, Umeå, Sweden

**Keywords:** Longitudinal, Risk factors, Sickness absence, Workers

## Abstract

**Purpose:**

This study aimed to identify sub-groups of workers with different trajectories of sick leave due to musculoskeletal pain over 1 year, and to investigate the extent to which the identified trajectories are associated with personal, occupational, lifestyle, and pain-related factors at baseline.

**Methods:**

Data on 981 blue- and white-collar workers were analyzed in the DPHACTO cohort (2012–2014). The number of days on sick leave due to pain was reported using text messages at 4-week intervals across 1 year. Latent class growth analysis was used to distinguish sub-groups with different trajectories of sick leave. A web-based questionnaire at baseline was used to assess personal, occupational (physical and psychosocial), lifestyle, and pain-related factors. Multinomial regression models were constructed to determine associations between baseline factors and trajectories of sick leave (referencing no sick leave), with adjustment for potential confounders.

**Results:**

Four distinct sub-groups were identified, with trajectories of sick leave due to pain ranging from no sick leave (prevalence 76%; average 0.5 days/year) to some days and increasing sick leave due to pain over 1 year (2%; 89 days/year). The increasing trajectory of sick leave was associated with higher perceived physical exertion, more time in manual work, less social community and influence at work, less leisure-time physical activity, smoking, and more severe symptoms (e.g., multisite pain, low back pain intensity, and pain interference).

**Conclusions:**

We identified four distinct trajectories of sick leave due to musculoskeletal pain. The sub-group with increasing sick leave due to pain was associated with several modifiable physical and psychosocial factors at work and outside work, which may have implications for prevention.

**Electronic supplementary material:**

The online version of this article (10.1007/s00420-019-01447-y) contains supplementary material, which is available to authorized users.

## Introduction

Sick leave due to musculoskeletal disorders (MSD) such as pain in the neck, shoulders, and back is common (Andersen et al. 2012; Lidwall 2015; Pekkala et al. 2018) and results in excessive economic costs both for organizations and society (Bevan 2015; Bhattacharya 2014). Identifying potentially modifiable determinants of sick leave due to MSD is an important step toward effective targeted interventions and prevention.

Patterns of sick leave can vary considerably between individuals and across time (Magee et al. 2016). For instance, a single episode of sick leave due to pain may consist of very few, some, or many consecutive days, and these episodes can re-occur more or less frequently over time. This variation may reflect distinct sub-groups with different trajectories of sick leave and different underlying causes (Feleus et al. 2016; Haukka et al. 2014; Magee et al. 2016). Thus, in addition to investigating the incidence of long-term sick leave, it is important to use an analytical approach that can capture the time course of sick leave in detail. Identifying sub-groups with different trajectories of sick leave due to musculoskeletal pain could be important for better understanding of determinants, optimizing prevention and reduction of sick leave due to MSD (Feleus et al. 2016; Haukka et al. 2014).

Sick leave is often of multifactorial origin and different occupational, lifestyle, and pain-related factors have been identified as predictors of sick leave due to pain in the previous studies (Andersen et al. 2012; Ariens et al. 2002; Feleus et al. 2016; Haukka et al. 2017; Holtermann et al. 2010; Lötters and Burdorf 2006), although with considerable differences in study designs, populations, and investigated factors. Higher rates of sick leave due to MSDs are found in workers with lower occupational class (Pekkala et al. 2018) and high physical work demands (Feleus et al. 2016; Holtermann et al. 2010; Lötters and Burdorf 2006). Heavy physical work was associated with a greater risk of sick leave from all causes in workers with neck–shoulder or back pain (Holtermann et al. 2010), and higher perceived workload was associated with an unfavorable prognosis of sick leave due to MSD (Lötters and Burdorf 2006). In agreement, low occurrence of exposure to lifting and repetitive physical work was associated with a favorable prognosis of sick leave from all causes among workers with multisite pain (Haukka et al. 2017). In contrast to heavy physical work, several prospective studies suggest that exercise and physical activity during non-work time (leisure) are protective against pain (Shiri and Falah-Hassani 2017; Steffens et al. 2016) and sick leave due to pain (van Amelsvoort et al. 2006). Furthermore, high pain intensity and multisite pain are potentially important determinants of a poor prognosis of sick leave (Haukka et al. 2013, 2014; Lötters and Burdorf 2006). However, while the studies referred to above suggest several important factors to sick leave, there is still limited research investigating potential modifiable determinants of group-based trajectories of sick leave due to musculoskeletal pain.

Some previous studies have distinguished sub-groups of workers with different trajectories of sick leave from all causes (Haukka et al. 2013, 2017; Magee et al. 2016) and due to musculoskeletal pain (Feleus et al. 2016; Haukka et al. 2014). While the two latter studies investigated predictors of sick leave due to pain, only one of them (Haukka et al. 2014) also included workers without pain. Including both asymptomatic workers and those with different MSDs is important to generalize results to the working population. Further knowledge on the occurrence, characteristics, and determinants of sick leave trajectories due to musculoskeletal pain is needed among working populations with different working conditions and symptom severities. Thus, this study was conducted among white- and blue-collar workers from three occupational sectors (cleaning, manufacturing, and transportation) with a large dispersion in occupational demands and musculoskeletal pain.

This longitudinal study aimed to identify sub-groups of workers with different trajectories of sick leave due to musculoskeletal pain over 1 year, and to investigate the extent to which the identified trajectories are associated with personal, occupational, lifestyle, and pain-related factors at baseline.

## Methods

### Study design

This is a prospective study using data from the Danish PHysical Activity Cohort with Objective measurements (DPHACTO). The study protocol of DPHACTO is described in detail elsewhere (Jørgensen et al. 2013). Data were collected from April 2012 to May 2014 in blue and white-collar workers from 15 workplaces in Denmark, representing three occupational sectors (cleaning, manufacturing, and transportation).

Baseline data collection consisted of a web-based questionnaire and health examination. Additional measures were obtained on daily physical activity and heart rate, which are presented in detail elsewhere (Hallman et al. 2017a, b, 2018). During follow-up, repeated measurements of self-reported sick leave were collected every 4th week over 1 year (14 waves in total) using text messages.

### Study population

Inclusion criteria were current employment at any of the 15 enrolled work places and taking part in the study both at baseline and follow-up. Exclusion criterion was providing less than two responses about sick leave during the 1-year follow-up.

In total, 2107 eligible workers were invited through a screening questionnaire, 1119 consented to participate at baseline, and 981 were included after responding to the baseline questionnaire and providing valid data on sick leave during follow-up. Of the 981 workers included in this study, 797 were blue-collar workers and 185 were administration workers (white-collar) at the same workplaces. The consenters to participate were similar to the non-consenters in demographic, occupational, and pain-related factors (Jørgensen et al. 2019).

All participants provided their written informed consent prior to participation. The present study was conducted according to the Declaration of Helsinki, approved by the Danish Data Protection Agency, and evaluated by the Regional Ethics Committee in Copenhagen, Denmark (H-2-2012-011).

### Assessment of baseline factors (potential determinants)

A broad range of personal, occupational (physical and psychosocial), lifestyle, and pain-related factors at baseline were selected as potential determinants of sick leave due to pain. These factors were selected a priori based on theoretical and empirical evidence of their relationship with musculoskeletal pain and sick leave. Most factors (see below) were assessed using the web-based questionnaire (Hallman et al. 2018).

#### Personal factors

Age was determined from the workers’ Danish civil registration numbers. Gender was determined by the question “Are you male or female?”. Body mass index (BMI, kg/m^2^) was calculated from objectively measured height and body weight.

#### Occupational physical and psychosocial factors

Occupational sector was determined using information from the companies about occupational sector (cleaning, manufacturing, or transportation) and a question about occupational class (administration or blue-collar), which resulted in a variable with four categories (cleaning, manufacturing, transportation, and administration). Seniority in the job (years) was determined using the question “For how long have you had the kind of occupation that you have currently?”

Perceived physical exertion at work was determined using the question “How physically demanding do you normally consider your present work?” with a ten-point scale ranging from 1 (not at all) to 10 (extremely demanding) modified from Borg (Borg 1998). Lifting and carrying, and pushing and pulling at work were assessed using two items from the Danish Work Environment Cohort Survey, DWECS (Tüchsen et al. 2006), e.g., “How much of your working time do you carry or lift?”. The six-point scale ranges from 1 (never) to 6 (almost all the time).

Psychosocial factors (influence and social community at work) were determined based on the Copenhagen Psychosocial Questionnaire, COPSOQ (Pejtersen et al. 2010). Influence at work (decision authority) was measured using two items (Chronbach’s *α* 0.62), i.e., “Can you influence the amount of work assigned to you?” and “Do you have influence on what you do at work?”. Social community at work was measured using two items (Chronbach’s *α* 0.77), i.e., “Is there good co-operation between the colleagues at work?” and “Is there a good collaboration between the management and the employees?”. The five-point scale ranges from 0 (never/hardly ever) to 4 (always) and a summary score of the two items was calculated (0–8) for each dimension, respectively.

#### Lifestyle

Self-reported vigorous leisure-time physical activity (LTPA) was measured using the question “How much time have you on average spent in vigorous exercise or competitive sports during the past year?” from the Danish Work Environment & Health study (Andersen et al. 2018). The four-point scale ranges from 1 (> 4 h/week) to 4 (never), which was reversed so that higher values indicate more time in LTPA. Smoking was assessed using the question “Do you smoke?”, with four alternatives dichotomized into two categories smoker (daily smoking) and non-smoker (occasionally smoking, used to smoke, never smoked). Alcohol intake (units/week) was assessed using the question “Do you drink alcohol? How many units did you drink last week?”.

#### Pain-related factors

Using self-report, we assessed the intensity, duration, and localization of pain in multiple body sites, as well as pain interference in physical and social activities, as previously described in detail (Hallman et al. 2018). In brief, pain in seven different body sites (neck/shoulders, elbows, hands/wrists, lower back, hips, knees, and feet/ankles) was assessed using modified questions from the Standardized Nordic questionnaire for the analysis of musculoskeletal symptoms (Kuorinka et al. 1987). For each body site, we determined the peak pain intensity (scale 0–10) during the past 3 months; the number of days with pain during the past year using six response categories merged into three categories (0–7 days, 8–90 days, and > 90 days); and the number of pain sites as determined by a pain intensity score > 2 for each site. Pain interference in two dimensions (interference with performance of demanding physical work, and interference with social activities) was measured using two items based on the SF-36 survey (Sullivan et al. 1995) with a modified 11-point scale from 0 (no impact) to 10 (completely prevented it).

#### Prospective assessment of sick leave (outcome)

Text messages (SMS) were administered every 4th week over 1 year using the commercial software “SMS-Track” (https://sms-track.com/) to obtain frequent repeated measures of self-reported sick leave due to musculoskeletal pain. The SMS was administered on Sundays with a reminder the following Monday. Sick leave was assessed using a single item from the validated Outcome Evaluation Questionnaire (Keefe et al. 1992): “Within the past month, how many days have you been absent from work due to pain in muscles or joints?” with a response scale ranging from 0 to 31 days. Self-reported sick leave demonstrates good test–retest reliability and sufficient convergent validity against records (Johns and Miraglia 2015).

### Statistical analyses

Latent class growth analysis (LCGA) was used to distinguish trajectories of sick leave due to musculoskeletal pain over 1 year using the Latent Gold software (version 5.1, Statistical innovations, Belmont, MA, USA). In brief, LCGA uses observed repeated measures data (in this case, sick leave) to estimate individual growth parameters (i.e., intercept and slope). Individuals are then assigned to homogenous sub-groups (latent classes) based on maximum posterior probabilities. The LCGA is constructed so that the variance is fixed within each class, while the variance between classes varies. Thus, the resulting trajectories are homogenous within each class and heterogeneous between classes (Jung and Wickrama 2008).

The LCGA models were constructed with time (14 waves across 1 year) as a continuous predictor and days on sick leave as the dependent variable. Poisson distribution was used to accommodate that sick leave was measured as counts (days/month). Missing data of sick leave were considered missing at random (MAR) and included in the models without imputations. Linear and quadratic models were constructed, but the quadratic models did not lead to better fit and were thus discarded. Consecutive (linear) LCGA models were constructed, each adding one more class to the preceding model (i.e., 1–10 class solutions). To identify an appropriate class solution, all models were evaluated based on fit statistics [Bayesian information criterion (BIC), entropy, and *p* value from the bootstrap log likelihood ratio test (BLRT)], growth parameters (intercept and linear slope), and clinical relevance (Nylund et al. 2007; van de Schoot et al. 2016). Thereafter, the trajectory classes from the chosen model were compared using descriptive statistics of a broad range of personal, occupational, lifestyle, and pain-related factors.

Associations between the selected predictors (i.e., personal, occupational, lifestyle, and symptom-related factors) and trajectory class of sick leave due to pain were determined using multinomial regression analysis with trajectory class as the dependent variable. Using the R software (R: A language and environment for statistical computing. R Foundation for Statistical Computing, Vienna, Austria, 2014). Two different models were constructed for each predictor: (1) unadjusted model and (2) model adjusting for the covariates age, gender, BMI, smoking, and occupational class (white- and blue-collar workers). The covariates were selected a priori based on theoretical assumptions and empirical findings of their association with working conditions, MSD and sick leave. As some of the identified classed contained rather few cases, which could introduce bias to the estimates, we applied Firth’s bias reduction with penalized maximum likelihood to the models (Firth 1993; Georg and Michael 2002). For each model, odds ratio (OR) with 95% confidence intervals (CI) was determined, and the level of significance was *p* < 0.05.

## Results

### Flow of participants and compliance to text messages

Of the eligible workers, 981 provided questionnaire data at baseline and at least two responses about sick leave days during follow-up, comprising the analyzed study sample. On average, the workers had 1.2 (SD 2.5) missing responses to SMS during follow-up, and 90% responded to 10 SMS or more (14 SMS in total). Of the 981 workers included in this study, 111 (11%) did not respond at the last follow-up (wave 14). Reasons for not responding were leaving the workplace (51%), actively withdrawing from the study (12%) and other reasons (36%) such as technical issues or maternity leave.

### Distinguished trajectories of sick leave due to musculoskeletal pain

**Fig. 1:**
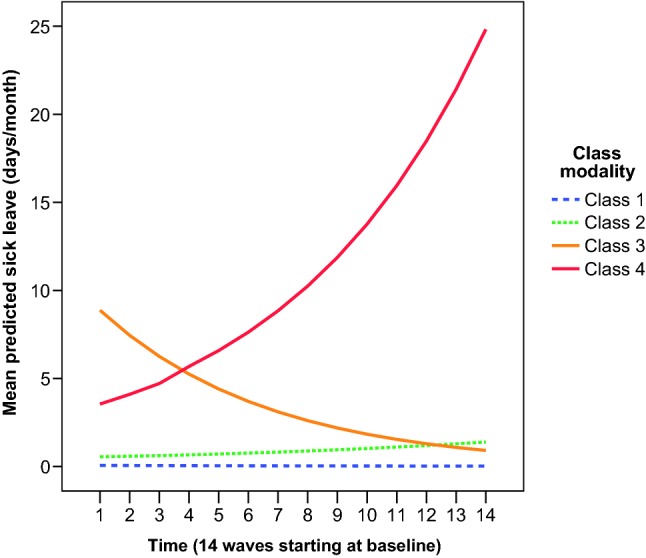
Distinguished trajectories of sick leave due to musculoskeletal pain over 1 year in the DPHACTO cohort (*n* = 981). The *x*-axis represents the repeated data collection every 4th week over 1 year starting at baseline (wave 1) with 14 waves in total. The *y*-axis represents the average predicted number of days on sick leave due to musculoskeletal pain the past month

Based on consecutive LCGA models with increasing number of classes, the 4-class trajectory solution was chosen based on model fit indices and clinical relevance (Table 1 and Fig. 1). The BIC values continued to improve (reduced values) for the 5-class and 6-class solutions, but entropy was reduced (Table 1), and the clinical distinction between the classes became less evident; that is, in the 5-class model, two classes had less than 1 day on sick leave per month over 1 year. The maximum posterior probabilities for the sick leave trajectories obtained in the 4-class solution are shown in supplement (Table S1).

**Table 1:** Model fit statistics for consecutive LCGA models with different number of classes

	2 classes	3 classes	4 classes	5 classes	6 classes
BIC	25,811	22,513	21,282	20,297	19,637
Entropy *R*^2^	0.99	0.95	0.94	0.91	0.91
BLRT *p* value	< 0.0001	< 0.0001	< 0.0001	< 0.0001	< 0.0001

BLRT is an inferential statistical test comparing a targeted class solution with a 1-less class solution; a significant BLRT value supports targeted class solution

BIC, Bayesian information criterion; BLRT, bootstrap likelihood ratio test; LCGA, latent class growth analysis

The four identified classes were characterized as follows (see Fig. 1):

Class 1 (“No sick leave”) had no sick leave days due to pain during 1 year (prevalence 76%).

Class 2 (“Few days—increasing trajectory”) had few sick leave days due to pain initially and a slow increase over 1 year (prevalence 19%).

Class 3 (“some days—decreasing trajectory”) had some sick leave days due to pain initially and a slow decrease over the year (prevalence 3%).

Class 4 (“Some days—increasing trajectory”) had some sick leave days due to pain initially and an increase over the year (prevalence 2%).

Table 2 shows the distribution of baseline characteristics and total days on sick leave due to pain across the four trajectory classes. The average number of reported days on sick leave due to pain during 1-year follow-up ranged from 0.5 days (class 1) to 89 days (class 4).

**Table 2:** Baseline characteristics across the four trajectory classes of sick leave due to musculoskeletal pain

	*N*	Class 1 (*n* = 746)	Class 2 (*n* = 186)	Class 3 (*n* = 27)	Class 4 (*n* = 22)
Personal					
Age (years)^a^	981	45 (10)	45 (9)	46 (9)	48 (13)
Female, %	981	47.7	50.0	55.6	50.0
BMI (kg/m^2^)^a^	956	27.2 (4.7)	27.7 (4.9)	29.6 (5.5)	26.1 (5.8)
Occupational					
Occupational sector, %	981				
Cleaning	172	15.8	21.0	33.3	27.3
Manufacturing	546	54.0	61.3	51.9	68.2
Transportation	79	7.8	10.2	7.4	0.0
Administration	184	22.4	7.5	7.4	4.5
Seniority (years)^a^	946	13.1 (10.2)	12.8 (10.3)	12.7 (8.1)	12.5 (12.0)
Physical exertion at work (scale 1–10)^a^	946	5.0 (2.5)	6.3 8 (2.1)	6.3 (2.2)	6.6 (1.9)
Pushing/pulling (scale 1–6)^a^	973	2.9 (1.5)	3.4 (1.5)	3.7 (1.7)	4.3 (1.6)
Lifting/carrying (scale 1–6)^a^	973	3.0 (1.5)	3.4 (1.6)	3.1 (1.4)	4.1 (1.4)
Influence at work (scale 0–8)^a^	747	5.3 (2.0)	5.1 (2.2)	5.5 (2.1)	4.2 (2.5)
Social community (scale 0–8)^a^	747	6.3 (1.3)	6.3 (1.3)	6.0 (1.6)	5.6 (1.4)
Lifestyle					
Self-reported vigorous LTPA (scale 1–4)^a^	942	2.6 (1.1)	2.5 (1.2)	2.4 (1.2)	1.7 (0.8)
Regular smoker, %	960	19.3	29.4	29.6	55.6
Alcohol intake (units/week)^a^	931	2.4 (6.1)	2.6 (6.5)	2.6 (4.7)	3.3 (5.6)
Pain characteristics					
Number of pain sites^a^	975	2.0 (1.7)	2.9 (1.8)	3.4 (2.1)	3.3 (1.9)
Pain duration (days past year), %	975				
0–7 days	246	29.1	14.6	7.4	4.5
8–90 days	387	40.8	38.4	40.7	13.6
> 90 days	342	30.1	47.0	51.9	81.8
NSP intensity (scale 0–10)^a^	975	3.1 (2.8)	4.1 (3.0)	4.7 (3.4)	4.7 (4.1)
LBP intensity (scale 0–10)^a^	975	2.9 (2.9)	4.3 (3.2)	5.3 (3.3)	5.8 (2.9)
Pain interference					
Physical work (scale 0–10)^a^	975	1.9 (2.6)	3.6 (3.0)	5.9 (3.1)	5.1 (2.8)
Social activities (scale 0–10)^a^	975	1.1 (2.1)	2.1 (2.6)	3.6 (3.3)	4.3 (3.4)
Sick leave due to pain (total days over 1 year)^a^	981	0.5 (1.0)	11.6 (8.5)	49.0 (26.7)	89.4 (75.9)

BMI, body mass index; LTPA, leisure-time physical activity; NSP, neck–shoulder pain; LBP, low back pain; class 1, no sick leave; class 2, few days—increasing trajectory; class 3: some days—decreasing trajectory; class 4: some days—increasing trajectory

^a^Mean (SD)

Based on descriptive comparisons (Table 2), the four sick leave trajectories due to pain were fairly similar in personal factors, while differences were seen in occupational, lifestyle and pain-related factors. For example, class 4 with some days increasing sick leave contained a larger proportion of manufacturing workers and smokers, and reported higher physical exertion at work, more time in manual work tasks (pushing/pulling and lifting/carrying), less influence and social community at work, less time in vigorous LTPA, and more severe symptoms compared to class 1 with no sick leave due to pain.

The adjusted regression models of associations between baseline factors and sick leave trajectories obtained from repeated measurements during 1-year follow-up are summarized below (Table 3). In all models, class 1 (no sick leave due to pain) was used as reference. The unadjusted models are shown in supplement (Table S2).

**Table 3:** Adjusted associations with trajectories of sick leave due to musculoskeletal pain (classes 1–4) for personal, occupational, lifestyle, and pain characteristics at baseline in the DPHACTO cohort (*N* = 981)

Predictors	Class 2		Class 3		Class 4	
	OR	95% CI	OR	95% CI	OR	95% CI
Personal						
Age (years)	0.99	0.98–1.01	1.01	0.97–1.05	1.03	0.98–1.09
Female (ref male)	1.09	0.78–1.53	1.23	0.57–2.70	1.09	0.42–2.89
BMI (kg/m^2^)	1.02	0.98–1.05	**1.09**	**1.02–1.17**	0.90	0.79–1.01
Occupational						
Blue-collar (ref administration)	**2.87**	**1.67–5.29**	2.31	0.72–11.66	2.37	0.55–22.12
Seniority (years)	1.00	0.98–1.02	0.99	0.95–1.03	0.98	0.92–1.03
Physical exertion at work (scale 1–10)	**1.23**	**1.13–1.35**	1.19	0.98–1.48	**1.30**	**1.01–1.72**
Pushing/pulling (scale 1–6)	**1.15**	**1.01–1.30**	**1.32**	**1.00–1.73**	**1.89**	**1.33–2.79**
Lifting/carrying (scale 1–6)	1.06	0.94–1.20	0.91	0.68–1.21	**1.73**	**1.24–2.48**
Influence at work (scale 0–8)	1.00	0.99–1.01	1.01	0.99–1.03	**0.98**	**0.96–1.00**
Social community (scale 0–8)	1.00	0.98–1.01	0.98	0.96–1.01	**0.97**	**0.94–1.00**
Lifestyle						
Vigorous LTPA (scale 1–4)	0.89	0.77–1.04	0.87	0.62–1.23	**0.45**	**0.26–0.74**
Regular smoker (ref no)	**1.59**	**1.08–2.33**	1.80	0.73–4.09	**4.34**	**1.65–12.03**
Alcohol intake (units/week)	0.98	0.95–1.01	0.93	0.82–1.02	1.01	0.92–1.07
Pain characteristics						
Number of pain sites (0–6)	**1.28**	**1.17–1.41**	**1.42**	**1.17–1.73**	**1.42**	**1.11–1.81**
Pain duration (ref 0–7 days)						
8–90 days	1.60	0.99–2.66	3.00	0.86–15.64	1.72	0.28–18.05
> 90 days	**2.92**	**1.81–4.83**	**4.49**	**1.32–23.24**	**7.83**	**1.82–73.13**
NSP intensity (scale 0–10)	**1.12**	**1.06–1.18**	**1.18**	**1.05–1.34**	1.06	0.90–1.24
LBP intensity (scale 0–10)	**1.15**	**1.09–1.21**	**1.24**	**1.10–1.40**	**1.28**	**1.10–1.51**
Pain interference						
Physical work (scale 0–10)	**1.21**	**1.15–1.29**	**1.49**	**1.31–1.71**	**1.36**	**1.16–1.60**
Social activities (scale 0–10)	**1.17**	**1.10–1.25**	**1.34**	**1.18–1.52**	**1.41**	**1.21–1.65**

Multinomial regression referencing class 1 (no sick leave due to pain) with adjustment for age, gender, BMI, smoking and occupational class (blue-collar; white-collar)

Significant (*p* < 0.05) associations are boldfaced

BMI, body mass index; LTPA, leisure-time physical activity; NSP, neck–shoulder pain; LBP, low back pain; class 1: no sick leave due to pain; class 2: few days—increasing trajectory; class 3: some days—decreasing trajectory; class 4: some days—increasing trajectory

#### Personal factors

Among the personal factors, only BMI was associated with trajectory class; that is, showing a statistically significant positive association with class 3 (some days decreasing sick leave due to pain). No significant association was found for age or gender with any sick leave trajectory (Table 3).

#### Occupational factors

Physical exertion at work showed significant positive associations with class 2 (few days increasing sick leave due to pain) and class 4 (some days increasing sick leave due to pain), and a non-significant positive association with class 3. Pushing/pulling showed significant positive associations with the three sick leave trajectories (classes 2–4), while lifting/carrying showed a significant positive association with class 4. Blue-collar work showed a significant positive association with class 2, and non-significant positive associations with class 3 and class 4. Psychosocial factors (influence and social community at work) showed significant negative associations with class 4 only (Table 3).

#### Lifestyle factors

Vigorous LTPA showed a significant negative association with class 4 and non-significant negative associations with class 2 and class 3. Current smoking showed significant positive associations with class 2 and class 4, while the positive estimate for class 3 was non-significant. Alcohol intake showed no significant association with any sick leave trajectory (Table 3).

#### Pain characteristics

As expected, most pain characteristics showed strong significant associations with sick leave trajectories. For instance, more pain sites, longer duration of pain, higher pain intensity in the lower back, and more interference of pain in physical work and social activities were associated with class 4 (Table 3).

## Discussion

This study identified sub-groups of workers with different 1-year trajectories of sick leave due to musculoskeletal pain, and investigated their associations with personal, occupational, lifestyle, and pain-related factors at baseline. We found four distinct trajectories of sick leave that were associated with several modifiable physical and psychosocial factors at work, and lifestyle and pain-related factors.

Of the four distinguished trajectories of sick leave due to pain, most of the workers were classified as “no sick leave” (76%) or “few days—increasing trajectory” (19%). Only modest proportions were classified as “some days—decreasing trajectory” (3%) or “some days—increasing trajectory” (2%). Due to differences in study populations and measures of sick leave across different studies, the occurrence of sick leave due to pain in this study cannot be directly compared with the previous studies on sick leave trajectories (Feleus et al. 2016; Haukka et al. 2013, 2014). Nevertheless, the 22 workers with some days and increasing sick leave due to pain (class 4) contributed with approximately 2000 days on sick leave over 1 year, which would be associated with a considerable loss in production and economic costs if applied to the overall working population (Bhattacharya 2014; Borghouts et al. 1999; Hansson and Hansson 2005). Thus, this sub-group of workers at increased risk of sick leave due to pain deserves special attention regarding prevention and treatment.

The LCGA allowed a detailed representation of sick leave trajectories at a group level that could not be captured by determining the incidence of sick leave or the total number of days absent for the individual. The identified sub-groups with distinct trajectories of sick leave provide novel information about the time course of sick leave due to pain and the potential determinants of this time course. Thus, our findings may inform research and practice about potential target groups at high risk of sick leave due to musculoskeletal pain that may need specific prevention strategies both at the occupational and individual levels.

As expected, the identified trajectories of sick leave due to pain differed in various pain characteristics at baseline. The adjusted regression models referencing no sick leave due to pain (class 1) showed that the other three trajectories (classes 2–4) were associated with more pain sites, longer pain duration, higher pain intensities, and more interference of pain in physical work and social activities. Interestingly, pain intensity was only predictive of “some days—increasing” sick leave due to pain (class 4) for the lower back, but not for the neck–shoulder region. In agreement, high pain intensity of the lower back was a major prognostic factor for sick leave duration from MSD in a previous study (Lötters and Burdorf 2006). This may suggest that the severity of low back pain is more important than neck–shoulder pain for the prognosis of sick leave due to pain. However, this should be further investigated by discriminating sick leave caused by different diagnoses of MSD. Multisite pain was associated with elevated risks of sick leave in several previous studies (Feleus et al. 2016; Haukka et al. 2013, 2014), which is supported by our findings. Overall, these findings indicate that information on various pain characteristics may be useful in identifying workers at risk of sick leave due to musculoskeletal pain, which could aid preventive efforts.

Among the personal factors at baseline, only BMI showed a positive association with “some days—decreasing” sick leave due to pain (class 3). Higher BMI has shown to be associated with an increased risk of musculoskeletal pain (Okifuji and Hare 2015) and sick leave (Neovius et al. 2008). Still, the nature of the observed relationship with BMI is unclear and likely explained by multiple factors acting mainly via indirect pathways. We found no association between age and sick leave trajectories, which might be explained by a healthy worker effect; that is, possible change to other jobs or early retirement among workers who develop prominent symptoms. The lack of association with gender is somewhat surprising, as the previous studies have found a higher prevalence of MSDs for women than men (Hogg-Johnson et al. 2009).

We found that several physical exposures at work were associated with sick leave trajectories. Compared with no sick leave due to pain (class 1), a one unit increment in perceived physical exertion at work (scale 1‒10) was associated with 30% increased likelihood of “some days—increasing” sick leave due to pain (class 4). Similarly, pushing/pulling, and lifting/carrying at work were both positively associated with “some days—increasing” sick leave. These results corroborate the previous studies indicating that high physical workloads increase the risk of a poor prognosis of all-cause and pain-related sick leave among workers (Andersen et al. 2016; Holtermann et al. 2010; Lötters and Burdorf 2006; Steenstra et al. 2005; Sundstrup et al. 2017). Thus, our findings support intervention strategies aiming at reducing high biomechanical exposures, such as pushing/pulling and lifting/carrying, in blue-collar work.

Reporting good social community and influence at work were associated with a reduced likelihood of “some days—increasing” sick leave due to pain, although the effects were small and marginally statistically significant. Still, this finding suggests that a good social community and a large extent of decision authority concerning work can, at least to some extent, be protective against sick leave due to MSD, which would be in agreement with some previous studies (Alavinia et al. 2009; Ariens et al. 2002; Karels et al. 2010).

Among the lifestyle factors, more time in vigorous LTPA was associated with a reduced likelihood of “some days—increasing” sick leave due to pain, while smokers showed an increased risk possibly due to other co-occurring behaviors and ill-health. The beneficial effect of LTPA on MSDs and sick leave is supported by several studies (Holtermann et al. 2012; Shiri and Falah-Hassani 2017; van Amelsvoort et al. 2006), and is likely explained by a combination of psychological and physiological benefits.

## Methodological discussion

The frequent repeated data of sick leave across 14 waves, and the high response rate to SMS, are an obvious strength, as it allowed capturing the course of sick leave due to musculoskeletal pain in detail. We also addressed a broad range of relevant predictors in a population of workers with a sufficient dispersion of exposure at work. This increases the possibility of identifying potentially important predictors of sick leave due to pain.

The results of this study must also be interpreted in relation to some potential limitations. First, the outcome sick leave due to pain was based on self-report, which is reasonably reliable and valid according to meta-analytical evidence (Johns and Miraglia 2015). Although there is a risk of underestimation of sick leave days (Fredriksson et al. 1998; Johns and Miraglia 2015; Stapelfeldt et al. 2012), we expect any bias to be marginal due to the short recall period of 1 month. Pain characteristics at baseline were strong predictors of sick leave due to musculoskeletal pain in this population of workers, and pain intensity predicted sick leave even on a monthly basis (Hallman et al. 2019). However, we did not investigate sick leave due to other causes than musculoskeletal pain, which is a potential limitation. In addition, the general question about sick leave due to pain did not distinguishing between different musculoskeletal diagnoses. It is possible that the determinants of sick leave due to musculoskeletal pain may differ between those with pain in the upper- and lower extremities (Lötters and Burdorf 2006), and thus, our estimates may not be representative.

Second, the follow-up period of 1 year was relatively short to obtain a sufficient number of cases that developed longer sick leave episodes. Thus, the sample sizes of the identified trajectories with more sick leave due to pain (classes 3–4) were relatively small, which reduces the statistical power. Thus, we could not perform extensive multivariate models to identify independent predictors in the multinomial regression models. Instead, we tested single factors while adjusting for selected covariates. However, this approach precluded inferences about the relative importance of different factors, as well as their possible interactions.

Third, physical exposure data were obtained using self-report, which has shown reduced precision and accuracy compared to objective measures (Gupta et al. 2016). Objective measures may have yielded different results. Furthermore, although we addressed a broad range of predictors at baseline, it is possible that changes occurred over time, which could have influenced the results. Thus, future studies would benefit from simultaneous assessment of exposures and outcome over time.

## Conclusion

Four distinct trajectories of sick leave due to musculoskeletal pain were identified among workers. The sub-group with some days and increasing sick leave over 1 year was associated with more severe symptoms and several modifiable physical and psychosocial factors at work and outside work, which may have implications for prevention and treatment. Future studies should use larger sample sizes and a longer follow-up while assessing exposure repeatedly over time, preferably using objective measures.

## Electronic supplementary material

Below is the link to the electronic supplementary material.

Supplementary material 1 (DOCX 28 kb)
