# Supplementary material for: Sick leave due to musculoskeletal pain: determinants of distinct trajectories over 1 year
Source: Int Arch Occup Environ Health. 2019 Jun 4;92(8):1099–108. doi: 10.1007/s00420-019-01447-y (PMC6814632; doi:10.1007/s00420-019-01447-y)
Supplement: Supplementary file 1 — Supplementary material 1 (DOCX 28 kb) [file 420_2019_1447_MOESM1_ESM.docx]

**Supplementary Material**

**Table S1.** Posterior probabilities (means) of class membership obtained from the 4-class LCGA.

| **Classes** | Class 1 | Class 2 | Class 3 | Class 4 |
| --- | --- | --- | --- | --- |
| Class 1 | **0.991** | 0.009 | 0.000 | 0.000 |
| Class 2 | 0.034 | **0.960** | 0.005 | 0.002 |
| Class 3 | 0.000 | 0.000 | **1.000** | 0.000 |
| Class 4 | 0.000 | 0.039 | 0.004 | **0.957** |

Abbreviation: LCGA, Latent Class Growth, Analysis.

**Table S2**. Unadjusted associations of personal, occupational and pain related factors at baseline with sick leave trajectories due to musculoskeletal pain. Multinomial regression referencing no sick leave due to pain (class 1).

|  | Class 2 |  | Class 3 |  | Class 4 |  |
| --- | --- | --- | --- | --- | --- | --- |
| Predictors | OR | 95%CI | OR | 95%CI | OR | 95%CI |
| **Personal** |  |  |  |  |  |  |
| Age (years) | 1.00 | 0.98 ‒1.02 | 1.01 | 0.97 ‒ 1.06 | 1.03 | 0.99 ‒1.08 |
| Female, % | 1.10 | 0.79 ‒1.51 | 1.36 | 0.64 ‒ 2.95 | 1.10 | 0.47 ‒2.54 |
| BMI (kg/m2) | 1.02 | 0.99 ‒1.05 | **1.10** | **1.02 ‒ 1.18** | 0.95 | 0.85 ‒1.05 |
| **Occupational** |  |  |  |  |  |  |
| Blue-collar (ref administration) | **3.44** | **2.02 ‒6.29** | 2.95 | 0.95 ‒ 14.64 | **4.14** | **1.06 ‒37.43** |
| Seniority (years) | 1.00 | 0.98 ‒1.01 | 1.00 | 0.96 ‒ 1.03 | 1.00 | 0.95 ‒1.04 |
| Physical exertion at work (scale 1-10) | **1.26** | **1.17 ‒ 1.36** | **1.26** | **1.06 ‒ 1.51** | **1.33** | **1.09 ‒ 1.68** |
| Pushing/pulling (scale 1-6) | **1.28** | **1.15 ‒1.41** | **1.43** | **1.12 ‒ 1.81** | **1.78** | **1.36 ‒2.36** |
| Lifting/carrying (scale 1-6) | **1.21** | **1.09 ‒1.34** | 1.06 | 0.81 ‒ 1.36 | **1.60** | **1.22 ‒2.10** |
| Influence at work (scale 0-8) | 1.00 | 0.99 ‒ 1.01 | 1.00 | 0.99 ‒ 1.02 | **0.98** | **0.96 ‒ 1.00** |
| Social community (scale 0-8) | 1.00 | 0.99 ‒ 1.01 | 0.98 | 0.96 ‒ 1.01 | **0.97** | **0.95 ‒ 1.00** |
| **Lifestyle** |  |  |  |  |  |  |
| Vigorous LTPA (scale 1-4) | **0.86** | **0.74 ‒0.99** | 0.85 | 0.61 ‒ 1.2 | **0.43** | **0.25 ‒0.69** |
| Regular smoker, % | **1.75** | **1.20 ‒2.52** | 1.82 | 0.75 ‒ 4.02 | **5.14** | **2.04 ‒13.32** |
| Alcohol intake (units/week) | 0.98 | 0.95 ‒1.01 | 0.93 | 0.82 ‒ 1.01 | 1.00 | 0.90 ‒1.06 |
| **Pain characteristics** |  |  |  |  |  |  |
| Number of pain sites (0-6) | **1.30** | **1.19 ‒1.42** | **1.49** | **1.23 ‒ 1.81** | **1.46** | **1.18 ‒1.80** |
| Pain duration (ref 0-7 days) |  |  |  |  |  |  |
| 8-90 days | **1.86** | **1.17 ‒3.03** | 3.29 | 0.96 ‒ 17.07 | 1.67 | 0.27 ‒17.32 |
| >90 days | **3.08** | **1.95 ‒4.99** | **5.62** | **1.7 ‒ 28.71** | **11.95** | **2.99 ‒108.56** |
| NSP intensity (scale 0-10) | **1.12** | **1.06 ‒1.19** | **1.20** | **1.06 ‒ 1.36** | **1.19** | **1.04 ‒1.37** |
| LBP intensity (scale 0-10) | **1.16** | **1.10 ‒1.22** | **1.27** | **1.13 ‒ 1.45** | **1.35** | **1.18 ‒1.56** |
| Pain interference |  |  |  |  |  |  |
| Physical work (scale 0-10) | **1.23** | **1.16 ‒1.30** | **1.53** | **1.35 ‒ 1.76** | **1.41** | **1.24 ‒1.62** |
| Social activities (scale 0-10) | **1.19** | **1.12 ‒1.27** | **1.39** | **1.23 ‒ 1.57** | **1.48** | **1.30 ‒1.70** |

Note: Significant (p<0.05) associations are boldfaced. Abbreviations: BMI, body mass index; LTPA, leisure-time physical activity; NSP, neck-shoulder pain; LBP, low back pain..

Class 1, No sick leave due to pain; Class 2, Few days – increasing trajectory; Class 3: Some days – decreasing trajectory; Class 4: Some days – increasing trajectory
